# Maternal anemia is associated with adverse maternal and neonatal outcomes in Mbarara, Uganda

**DOI:** 10.1080/14767058.2023.2190834

**Published:** 2023-12

**Authors:** P. Kaitlyn Edelson, Danni Cao, Kaitlyn E. James, Joseph Ngonzi, Drucilla J. Roberts, Lisa M. Bebell, Adeline A. Boatin

**Affiliations:** a Department of Obstetrics and Gynecology, University of Pennsylvania, Philadelphia, PA, USA; b Brandeis University, Waltham, MA, USA; c Department of Obstetrics and Gynecology, Massachusetts General Hospital, Boston, MA, USA; d Department of Obstetrics and Gynaecology, Mbarara University of Science and Technology, Mbarara, Uganda; e Department of Pathology, Massachusetts General Hospital, Boston, MA, USA; f Infectious Diseases Division, Massachusetts General Hospital, Boston, MA, USA; g Center for Global Health, Massachusetts General Hospital, Boston, MA, USA

**Keywords:** Anemia, blood transfusion, global health, maternal morbidity, neonatal morbidity, placental pathology

## Abstract

**Purpose:**

Maternal anemia is a significant risk factor for maternal morbidity and mortality, increasing risk of preterm birth, intrauterine growth restriction, stillbirth, and death. Moderate and severe anemia in pregnancy is defined as hemoglobin (Hb) <10 g/dl and Hb < 7 g/dl, respectively. We aimed to characterize the association of maternal anemia with maternal, neonatal, and placental outcomes in a resource-limited setting.

**Methods:**

Data were collected from a prospective cohort of 352 pregnant women at a tertiary academic Ugandan hospital. One hundred and seventy-six (50%) of women were living with HIV. Hemoglobin was measured in labor, and placentas were collected postpartum. Maternal outcomes included mode of delivery, hemorrhage, blood transfusion, intensive care unit admission, and maternal mortality. Neonatal outcomes included gestational age at delivery, birthweight, stillbirth, and neonatal mortality. Placental descriptors included weight and thickness. Categorical variables were analyzed using Chi-squared and Fisher’s exact tests.

**Results:**

Hemoglobin < 10 g/dl, was present in 17/352 (5%) of women. Significantly more women with moderate or severe anemia were HIV-infected: 14/17 (82%) versus 162/335 (48%) (*p* = .006). Blood transfusions (2/17, 12% versus 5/335, 2%, *p* = .04) and neonatal deaths (2/17, 12% versus 9/335, 3%, *p* = .01) were more common in the anemia group. Placental thickness was lower in the anemia group (1.4 cm versus 1.7 cm, *p* = .04).

**Conclusions:**

Moderate and severe anemia was associated with maternal HIV infection, maternal blood transfusion, neonatal death, and decreased placental thickness. The overall rate of moderate and severe anemia among this cohort was lower than previously reported.

## Introduction

Maternal anemia is a significant and potentially modifiable risk factor for maternal morbidity and mortality, contributing to more than 115,000 maternal deaths globally per year [1]. The World Health Organization (WHO) defines moderate and severe anemia in pregnancy as hemoglobin (Hb) <10 g/dl and Hb < 7 g/dl, respectively [2]. The burden of maternal anemia is high in low- and middle-income countries (LMICs), where prevalence among pregnant women is estimated between 35% and 60%, whereas the prevalence in high income countries is <20% [3,4]. Although prevalence of maternal anemia in high income countries has decreased over the years, the prevalence of maternal anemia in LMICs has remained almost the same since 2000 [4].

The causes of maternal anemia vary by region, though the most important contributors in LMICs are iron deficiency in the setting of chronic malnutrition, parasitic infection, and short inter-pregnancy intervals [4]. Pregnant women with anemia are at increased risk of preterm birth, small-for-gestational age babies, and all-cause perinatal death [4]. Antenatal anemia is also an underlying factor in morbidity and mortality due to postpartum hemorrhage, the most common cause of maternal mortality globally, contributing to one-third of maternal deaths in LMICs [5].

Even in the context of mild maternal anemia, placental adaptation has been described, including increased expression of angiogenic proteins [6,7], and increase in terminal villi blood vessels [8]. It has been hypothesized that these placental changes in the face of maternal anemia may mediate the poor fetal outcomes associated with maternal anemia, specifically intrauterine growth restriction and stillbirth.

While it is not clear that antenatal anemia is an independent risk factor for peripartum hemorrhage, the maternal morbidity and mortality consequences from peripartum hemorrhage in a mother who is already anemic, especially in contexts where ready access to safe blood transfusion may not be available, are more severe. We aimed to characterize the effect of moderate and severe maternal anemia on maternal, neonatal, and placental outcomes in a resource-limited setting. We also included placental examination in our analysis to investigate a potential relationship between placental characteristics and neonatal outcomes in the face of maternal anemia.

## Materials and methods

We performed a secondary analysis of data collected prospectively from a cohort of 352 pregnant women recruited from Mbarara Regional Referral Hospital (MRRH) in Uganda between September 2017 and February 2018 [9]. MRRH serves a mixed urban-agrarian population of nine million people and reports approximately 11,000 deliveries annually. All pregnant women presenting to MRRH in labor were screened for enrollment and considered eligible if they were 18 years of age or older, spoke English or Runyankole (local language), reachable by phone after discharge for follow-up, and not suspected to have multiple-gestation pregnancy. Women living with HIV (WLWH) meeting these criteria were eligible only if they reported taking antiretroviral therapy within the last 30 days.

The primary study was powered to detect a three-fold difference in chronic placental inflammation by maternal HIV serostatus. Thus, eligible WLWH were enrolled consecutively, and HIV-uninfected comparators were selected as the next eligible laboring woman presenting to MRRH after each enrolled WLWH. After 150 participants were enrolled, HIV-uninfected women were selectively enrolled to balance parity and gestational age by HIV serostatus. The study was approved by the institutional ethics review boards at Mbarara University of Science and Technology (MUST, 11/03–17), Partners Healthcare (2017P001319/MGH), and the Uganda National Council of Science and Technology (HS/2255). Hemoglobin was measured for all enrolled women using point-of-care Hb estimation on blood collected during labor via peripheral venipuncture. Point-of-care malaria (Standard Diagnostics, Yongin-si, South Korea), syphilis (Standard Diagnostics, Yongin-si, South Korea), and HIV (Determine HIV 1/2, Abbott, Abbott Park, IL, HIV-uninfected women only) testing was also conducted on venipuncture blood.

Maternal demographics, obstetric and medical history, and maternal and neonatal outcomes were determined through chart review and in-person questionnaires administered. Maternal outcomes examined included Hb at delivery, mode of delivery, peripartum or postpartum hemorrhage, blood transfusion, transfer for intensive care unit, and maternal mortality. Neonatal outcomes examined included gestational age at delivery, birthweight, umbilical cord Hb level, stillbirth, and in-hospital neonatal mortality.

Placental descriptors included placental weight, placental thickness, and placental histology. Placentas were collected at birth for gross and histologic examination. Placentas were examined in the fresh state by trained research assistants. Standard measurements were obtained including umbilical cord length, two diameters of the placenta and the maximal placental thickness, as well as the trimmed placental weight. Three routine sections were obtained on every placenta including a section of umbilical cord and a membrane roll and two full thickness sections of parenchyma. Additional sections were taken from any grossly identified lesion. Sections were formalin fixed and processed routinely at MUST, then slides were sent for histopathologic review by an experienced perinatal pathologist (DJR) blinded to the clinical history and gross findings. Histologic diagnoses followed the Amsterdam consensus recommendations [10].

Using the WHO definition of moderate or severe anemia for pregnancy (Hb < 10 g/dl), we stratified our cohort by Hb level measured during labor. We compared maternal, neonatal and placental outcomes in women with moderate or severe anemia and women with normal Hb levels. Categorical variables were analyzed using Fisher’s exact tests; continuous variables were analyzed using a *t*-test. Data analysis was performed using Stata 15 (StataCorp, College Station, TX).

## Results

During the study period, a total of 1940 potential subjects were approached for potential enrollment. Of these, 451 did not meet inclusion criteria; of these, only seven potential subjects were excluded due to lack of antiretroviral therapy. Of the 1489 subjects meeting inclusion criteria, 1135 were not enrolled, largely due to the fact that they were not the “next” eligible subject after a WLWH was enrolled, leaving 352 subjects enrolled and available for analysis; CONSORT diagram available in primary publication [9]. Of these, 176 (50%) were WLWH. Maternal demographic data and infectious co-morbidity data are presented in Table 1. The mean age in the cohort was 26.5 years (±0.3). Most participants 317/352 (90%) were married, with a median parity of 3. About half the participants had completed primary school (145/352 (41.2%)) and about half (223/352 (63.3%)) attended four or more antenatal visits.

The mean Hb level in the cohort was 12.7 g/dl (±0.09). A total of 17/352 (5%) participants met criteria for moderate or severe anemia. There were no statistically significant differences between the Hb < 10 g/dl group and the Hb ≥ 10 g/dl group in terms of age, parity, household income, years of education, or number of antenatal care visits attended. There were significantly more married women in the non-anemic group (305/335, 91%) compared to the anemic group (12/17, 70%, *p* = .006). There were no differences in prevalence between malaria and syphilis groups, though the study was insufficiently powered to detect such a difference. The rate of malaria in the anemic group was 5.9% compared to 12.2% in the non-anemic group; the rate of syphilis was 0% in the anemia group compared to 3.9% in the non-anemia group. Rates of micronutrient supplementation with folate and iron were similar between groups, with 11/17 (64.7%) supplement use in the anemic group compared to 231/335 (69.0%) in the non-anemic group. There was only one subject who reported tobacco use, in the non-anemic group. HIV prevalence differed significantly by anemia group status, with 14/17 (82%) anemic women and 162/335 (48%) nonanemic WLWH (*p* = .006).

The mean Hb level was 8.5 g/dl in the anemic group, significantly lower than 13.0 g/dl in the non-anemic group (*p* = .001). There were no statistically significant differences between the groups in terms of gestational age at delivery, proportion delivering via cesarean, postpartum hemorrhage, ICU transfer, peripartum fever, or maternal death (Table 2). Despite similar prevalence of peripartum or postpartum hemorrhage between the groups, anemic women received significantly more blood transfusions than non-anemic women (2/17, 11.8% versus 5/335, 1.5%, *p* = .004). The obstetric outcomes were then stratified by HIV status, and there were no differences, with the exception of significantly more anemic WLWH receiving blood transfusions (2/14, 14.3%) compared to anemic women without HIV (0/3, 0%) (see Supplemental Table 1).

Neonatal outcomes were similar between groups, including birthweight, umbilical cord Hb level, APGAR score, and antenatal or intrapartum stillbirth (Table 3). There were significantly more in-hospital neonatal deaths in the anemic group than the non-anemic group (2/17, 11.8%, versus 9/335, 2.7% *p* = .04). Placental weights and number of focal placental lesions did not differ between the anemic and non-anemic groups, though placental thickness was significantly greater in the non-anemic group (1.7 cm versus 1.4 cm, *p* = .04). There were no differences in the histopathologic findings between the groups. Neonatal and placental outcomes were then stratified by HIV status, and no differences in outcomes were detected by HIV status (see Supplemental Table 2).

## Conclusions

In this secondary analysis of a prospective cohort of women delivering in southwestern Uganda, we found a 5% overall prevalence of moderate and severe anemia, notably lower than what has been previously reported in similar global health settings [11,12]. There were no significant differences in risk of preterm birth, growth restriction, peripartum or postpartum hemorrhage or maternal death between anemic and non-anemic laboring women.

Despite similar rates of peripartum or postpartum hemorrhage among anemic and non-anemic women, we did observe a significant difference in the proportion receiving blood transfusion (11.8% of anemic women versus 5/335 of non-anemic women, *p*= .004). This finding is consistent with prior work identifying maternal anemia as an independent risk factor for transfusion at the time of delivery, regardless of delivery blood loss [13]. A recent cohort study by Smith et al. found that women with antenatal anemia had a 2.45-fold increased risk of blood transfusion in the intrapartum and postpartum period compared to women without anemia [14]. This finding has implications for resource utilization, specifically in resource-limited settings where blood transfusion may not be reliably available in a timely fashion. Thus, decreasing need for transfusion by optimizing antepartum maternal Hb may be an important strategy to decrease transfusion needs.

In the context of our study population, which is enriched for WLWH taking antiretroviral therapy, it is challenging to attribute the cause of anemia. Both HIV infection, as well as therapy for this infection, can contribute to increased prevalence of anemia [15]. However, this study included only WLWH who were taking antiretroviral therapy, and the most common regimens of antiretroviral therapy (TDF/3TC/EFV and AZT/3TC/EFV making up >80% of the regimens [9]) are not associated with significant anemia; thus the effect of either HIV or antiretroviral therapy for HIV does not appear to impact of anemia in our cohort.

Consistent with our findings, multiple studies have shown an association between neonatal death and moderate or severe maternal anemia [16,17]. Associations between maternal anemia and poor neonatal outcomes including preterm birth, low birthweight, and stillbirth have also previously been reported [14,18]. We did not find the same associations with preterm birth, low birthweight, or stillbirth in our cohort, possibly due to epidemiologic differences in this setting, or smaller sample size than prior studies. However, the proposed mechanism by which maternal anemia results in preterm birth and low birthweight is through placental insufficiency, and we did find that anemic women had significant lower placental thickness than non-anemic women, possibly reflecting an increased prevalence placental insufficiency among anemic women. The relationship between maternal anemia and placental thickness is not clearly defined in the literature, but some proposed mechanisms include iron deficiency promoting pro-inflammatory cytokines, dysregulation of placental angiogenesis, and decrease in placental insufficiency, resulting in altered placental morphology [19]. Some studies of placental morphology have suggested a relationship between placental disk thickness and growth restriction [20], though other studies have not found this association [21].

Strengths of this study include the prospective nature of the study, relatively large cohort size, and little missing data. The distribution of known potential confounders for maternal anemia (malaria, income, parity, education attainment) were equally distributed between the groups, reducing the risk of confounding bias. Importantly, this study associates placental gross pathology findings with maternal anemia, pointing to a possible mechanism by which maternal anemia may be associated with neonatal outcomes. Placental gross pathology data have rarely been included in studies of maternal anemia done in similar settings.

Weaknesses of this study include the design as a secondary analysis of a cohort enriched for WLWH taking antiretroviral therapy. This raises the specter of possible selection bias, as women already taking a daily medication may be more likely to seek care, receive antenatal Hb testing, and take iron supplements prior to delivery. Our ability to adjust for potential confounders was limited by sample size, which precluded regression analysis. Additionally, the cohort was recruited from a tertiary referral center which may attract women with a higher socioeconomic status (SES). Prior studies have shown associations between higher SES, higher antenatal Hb, and better nutritional status [22]. These potential selection and population biases may limit the generalizability of our findings in resource-limited settings. Additionally, we measured maternal Hb at the time of presentation for delivery, which does not necessarily reflect maternal anemia during the critical portions of the second and third trimester. Finally, the low prevalence of moderate to severe anemia with Hb < 10 g/dl (5% of our cohort) limits power to detect differences in rare maternal and neonatal outcomes such ICU transfer and maternal mortality.

Treating maternal anemia can be very challenging even in high-resource contexts. As iron deficiency is the most common cause of maternal anemia [4], standard therapy for treatment of anemia in pregnancy, as recommended by the WHO, is 60–120 mg oral (PO) iron daily [23]. Future research studies should investigate whether correcting antepartum maternal anemia prior to delivery reduces the risk of adverse maternal and neonatal outcomes. If so, future work should aim to better understand the etiology of maternal anemia in diverse contexts so that clinicians and public health practitioners can recommend effective and appropriate therapies to optimize antenatal maternal Hb levels.

In conclusion, antenatal moderate and severe anemia is associated with increased rate of adverse maternal, neonatal, and placental outcomes.

## Supplementary Material

Supplemental Tables

## Figures and Tables

**Table 1.** Maternal demographics.

	Hb < 10g/dl, *n*= 17	Hb ≥ 10g/dl, *n*= 335	*p* Value

Age in years, mean (SD)	27.0 (5.2)	26.5 (5.7)	.74
Parity, median (IQR)	3 (3–4)	3 (2–4)	.68
Married, *n* (%)	12 (70.6%)	305 (91.0%)	.02
Monthly income in US dollars, mean (SD)	69.22 (39.83)	62.64 (68.93)	.79
Completed at least primary school, *n* (%)	9 (52.9%)	136 (40.6%)	.80
Attended 4 or more antenatal care visits, *n* (%)	9 (52.9%)	214 (63.9%)	.44
HIV-positive, *n* (%)	14 (82.4%)	162 (48.4%)	.01
Malaria rapid diagnostic test positive, *n* (%)	1 (5.9%)	41 (12.2%)	.71
Syphilis rapid diagnostic test positive, *n* (%)	0 (0.0%)	13 (3.9%)	1.00
Micronutrient supplementation (folate, iron), *n* (%)	11 (64.7%)	231 (69%)	.79
Tobacco use, *n* (%)	0 (0.0%)	1 (0.3%)	1.00

**Table 2.** Obstetric outcomes.

	Hb < 10 g/dl, *n*= 17	Hb ≥ 10g/dl, *n*= 335	*p* Value

Hb (g/dl), mean (SD)	8.5 (1.2)	13.0 (1.4)	<.001
Gestational age at delivery weeks, mean (SD)	39.0 (1.7)	38.9 (3.6)	.86
Cesarean delivery, *n* (%)	7(41.2%)	107 (31.9%)	.43
Post-partum or peripartum-hemorrhage (PPH), *n* (%)	0 (0.0%)	11 (3.3%)	.43
Blood transfusion, *n* (%)	2 (11.8%)	5 (1.5%)	.004
Intensive care unit transfer, *n* (%)	0 (0.0%)	0 (0.0%)	–
Postpartum fever, *n* (%)	0 (0.0%)	1 (0.3%)	.96
Maternal death, *n* (%)	0 (0.0%)	0 (0.0%)	–

**Table 3.** Neonatal and placental outcomes.

	Hb < 10 g/dl, *n*= 17	Hb ≥ 10g/dl, *n*= 335	*p* Value

Birthweight (g), mean (SD)	3112 (492)	3208 (447)	.39
Umbilical cord Hb (g/dl), mean (SD)	15.2 (1.5)	15.1 (2.1)	.98
1-minute APGAR, mean (SD)	7.9 (2.7)	8.3 (1.6)	.46
5-minute APGAR, mean (SD)	8.7 (2.8)	9.5 (1.5)	.05
Stillbirth	1 (5.9%)	6 (6.8%)	.24
Neonatal death	2 (11.8%)	9 (2.7%)	.04
Placental weight (g)	444 (85)	458 (101)	.58
Placental thickness (cm)	1.4 (0.6)	1.7 (0.5)	.04
