# Supplementary material for: Maternal anemia is associated with adverse maternal and neonatal outcomes in Mbarara, Uganda
Source: J Matern Fetal Neonatal Med. Author manuscript; Available in PMC 2023 Dec 1. (PMC10419325; doi:10.1080/14767058.2023.2190834)
Supplement: Supplemental Tables [file NIHMS1910452-supplement-Supplemental_Tables.docx]

**Supplemental Table 1: Obstetric Outcomes Stratified by HIV Status**

|  | ***HIV Negative (n=176)*** | | | ***HIV Positive (n=176)*** | | |
| --- | --- | --- | --- | --- | --- | --- |
|  | **Hb<10 g/dl**  ***n*=3** | **Hb≥10 g/dl**  ***n*=173** | ***P*-value** | **Hb<10 g/dl**  ***n*=14** | **Hb≥10 g/dl**  ***n*=162** | ***P*-value** |
| Hb (g/dl), mean (SD) | 9.1 (0.6) | 13.2 (1.4) | <0.001 | 8.4 (1.3) | 12.7 (1.4) | <0.001 |
| Gestational age at delivery weeks, mean (SD) | 39.7 (1.5) | 38.9 (3.4) | 0.72 | 38.89286 (1.72246) | 38.78889 (3.707789) | 0.92 |
| Cesarean delivery, n (%) | 2 (66.7%) | 46 (26.6%) | 0.18 | 5 (35.7%) | 61 (37.7%) | 1.00 |
| Post-partum or peripartum-hemorrhage (PPH), n (%) | 0 (0.0%) | 6 (3.5%) | 0.73 | 0 (0.0%) | 5 (3.1%) | 0.49 |
| Blood transfusion, n (%) | 0 (0.0%) | 3 (1.7%) | 1.00 | 2 (14.3%) | 2 (1.2%) | 0.03 |
| Intensive Care Unit transfer, n (%) | 0 (0.0%) | 0 (0.0%) | -- | 0 (0.0%) | 0 (0.0%) | -- |
| Postpartum fever, n (%) | 0 (0.0%) | 1 (0.6%) | 0.37 | 0 (0.0%) | 0 (0.0%) | -- |
| Maternal death, n (%) | 0 (0.0%) | 0 (0.0%) | -- | 0 (0.0%) | 0 (0.0%) | -- |

**Supplemental Table 2: Neonatal and Placental Outcomes, Stratified by HIV Status**

|  | ***HIV Negative (n=176)*** | | | ***HIV Positive (n=176)*** | | |
| --- | --- | --- | --- | --- | --- | --- |
|  | **Hb<10 g/dl**  ***n*=3** | **Hb≥10 g/dl *n*=173** | ***P*-value** | **Hb<10 g/dl**  ***n*=14** | **Hb≥10 g/dl**  ***n*=162** | ***P*-value** |
| Birthweight grams, mean (SD) | 3233 (451) | 3259 (464) | 0.92 | 3085 (513) | 3152 (422) | 0.58 |
| Umbilical cord Hb (g/dl), mean (SD) | 14.4 (0.85) | 15.6 (1.97) | 0.30 | 15.4 (1.56) | 14.7 (2.1) | 0.26 |
| 1-minute APGAR, mean (SD) | 6.67 (4.0) | 8.3 (1.66) | 0.11 | 8.2 (2.4) | 8.2 (1.6) | 0.98 |
| 5-minute APGAR, mean (SD) | 8 (3.5) | 9.6 (1.3) | 0.052 | 8.9 (2.8) | 9.5 (1.7) | 0.25 |
| Stillbirth | 0 (0.0%) | 3 (1.7%) | 0.32 | 1 (7.1%) | 3 (1.9%) | 0.20 |
| Neonatal death | 1 (33.3%) | 4 (2.3%) | 0.09 | 1 (7.1%) | 5 (3.1%) | 0.40 |
| Placental weight grams | 0.46 (0.11) | 0.44 (0.07) | 0.66 | 0.45 (0.09) | 0.45 (0.09) | 0.86 |
| Placental thickness cm | 1.74 (0.54) | 1.2 (0.82) | 0.09 | 1.65 (0.55) | 1.46 (0.52) | 0.20 |

**Supplemental Table 3: Logistic Regression for Birthweight <2500 grams**

|  | OR (95% CI) | p-value |
| --- | --- | --- |
| *Unadjusted model* |  |  |
| Moderate to severe anemia | 5.25 (1.34, 20.6) | 0.017 |
|  |  |  |
| *Adjusted model* |  |  |
| Moderate to severe anemia | 5.15 (1.26, 21.0) | 0.023 |
| HIV status |  |  |
| HIV positive | 1.07 (0.37, 3.05) | 0.904 |
| HIV negative | Ref |  |
